# Influence of decompression by laminotomy and percutaneous tansforaminal endoscopic surgery on postoperative wound healing, pain intensity, and lumbar function in elderly patients with lumbar spinal stenosis

**DOI:** 10.1080/07853890.2025.2472865

**Published:** 2025-03-03

**Authors:** Haiyang Zhu, Yuan Liu, Yijing Wang, Denghui Xu, Zhe Zhao, Xuejian Wu

**Affiliations:** a Emergency Surgery, The First Affiliated Hospital of Zhengzhou University, Zhengzhou, China; b Imaging and Nuclear Medicine Ward, The First Affiliated Hospital of Zhengzhou University, Zhengzhou, China; c Department of Orthopedics, The First Affiliated Hospital of Zhengzhou University, Zhengzhou, China

**Keywords:** Lumbar spinal stenosis, decompression by laminotomy, percutaneous tansforaminal endoscopic surgery, postoperative wound healing, pain intensity, lumbar function, elderly patients

## Abstract

**Purpose:**

To compare the wound healing, pain intensity and lumbar function in elderly patients with lumbar spinal stenosis after laminectomy decompression or percutaneous transforaminal endoscopic surgery.

**Methods:**

A retrospective study was conducted on 65 patients who underwent laminotomy and 69 patients who underwent percutaneous transforaminal endoscopic spinal decompression surgery. clinical data analysis, including surgical parameters, complications, postoperative wound healing, pain intensity, lumbar function, and correlation analysis, was performed.

**Results:**

The operative time of percutaneous transforaminal endoscopic surgery was significantly shorter than that of laminotomy (70.78±6.80 min vs 128.97±4.70 min, *p* < 0.001), intraoperative blood loss was significantly reduced (94.22± 7.69ml vs 327.68± 6.44ml, *p* < 0.001), postoperative wound healing time and time to get out of bed were significantly shortened, pain was reduced by visual analog scale (3.48±1.11 vs 2.80±1.05, *p* = 0.007), the Japanese Orthopaedic Association (JOA) score was significantly increased, and the Oswestry Disability Index (ODI) score showed significantly decrease. The incidence of urinary tract infection and urinary retention was higher after laminotomy. Correlation analysis showed that operative time, intraoperative blood loss, and time to get out of bed were significantly related to prognosis in elderly patients.

**Conclusion:**

Percutaneous transforaminal endoscopic surgery is significantly superior to conventional laminectomy decompression in the treatment of elderly lumbar spinal stenosis.

## 1. Introduction

As individuals age, degenerative changes such as intervertebral disc and facet joint degeneration, hypertrophy of the ligamentum flavum, and spinal instability gradually lead to lumbar spinal sten osis [1,2]. Degenerative lumbar spinal stenosis (DLSS) was increasingly prevalent in the elderly population, characterized by symptoms of lumbar and leg pain, lower limb numbness, and intermittent claudication, significantly impacting patients’ walking ability, physical function, and quality of life [3–5]. In cases where conservative treatments fail to alleviate symptoms, surgical intervention becomes necessary [6]. Laminotomy decompression surgery can selectively alleviate pressure within the spinal canal, minimizing damage to the lumbar spine structure [7,8]. The appropriate window size was selected based on the severity of the patient’s symptoms and the size and location of the lesions [9]. However, due to the limited operative scope, patients with severe spinal canal stenosis may pose increased intraoperative challenges with insufficient decompression [9,10]. Nonetheless, this approach was associated with greater trauma and slower recovery, which may not be conducive to favorable patient outcomes [11]. With advancements in medical technology, minimally invasive surgery, such as percutaneous transforaminal endoscopic surgery, has gradually penetrated clinical practice [11,12]. This minimally invasive approach offers benefits such as reduced trauma and favorable outcomes, leading to its increasing adoption in clinical settings [13]. However, comparative reports on the application of laminotomy decompression and percutaneous transforaminal endoscopic treatment in elderly patients with lumbar spinal stenosis were relatively limited [6,14]. Therefore, this study aims to investigate the impact of laminotomy decompression and percutaneous transforaminal endoscopic treatment on postoperative wound healing, pain intensity, and lumbar function in elderly patients with lumbar spinal stenosis [15].

## 2. Materials and methods

### 2.1. Study population and grouping criteria

This retrospective study analyzed the clinical data of elderly patients with lumbar spinal stenosis admitted to our hospital from January 2021 to June 2023.The study included 65 patients who underwent laminotomy and 69 patients who underwent percutaneous transforaminal endoscopic spinal decompression surgery.

### 2.2. Inclusion and exclusion criteria

**Inclusion criteria:** Definitive diagnosis of lumbar spinal stenosis; age greater than or equal to 60 years; clinical symptoms, signs, and imaging examinations (X-ray, CT, and MR) are consistent; ineffective non-surgical treatment for more than 3 months or recurrent attacks; dynamic lumbar spine X-ray indicating good segmental stability; absence of prior lumbar spine surgical treatment in patients.

**Exclusion criteria:** Short course of spinal stenosis, mild symptoms; concomitant with intervertebral instability, fractures, spondylolisthesis, intervertebral infection, tuberculosis, tumors, or deformities; presence of segmental instability in lumbar spine disorders such as minor facet joint dislocation; concomitant severe medical conditions incompatible with surgery.

### 2.3. Preoperative preparation

Prior to surgery, all patients underwent thorough spinal MRI, X-rays, and CT scans. Detailed explanations of the surgical principles, procedures, and precautions were provided to both groups of patients. The assessment of patients’ physical condition and the functionality of vital organs such as the heart, lungs, liver, and kidneys aimed to evaluate their tolerance to surgery and intraoperative anesthesia. Hypertensive patients received antihypertensive medications preoperatively to adjust their blood pressure, aiming for an ideal blood pressure of 120-130/70-80 mmHg, not exceeding 150/90 mmHg. Routine evaluation of cardiac function and medication management, based on cardiology consultation, was conducted for patients with heart disease, implementing surgery on the basis of Grade 1 heart function. Diabetic patients underwent preoperative insulin therapy to control blood sugar levels.Patients with a history of cerebrovascular disease, coronary heart disease, or deep vein thrombosis underwent preoperative imaging to exclude any new lesions, following the relevant department’s consultation advice. Anticoagulant drugs were avoided around the perioperative period, and long-term oral clopidogrel users discontinued the medication one week before surgery, switching to subcutaneous injection of low-molecular-weight heparin. Patients with poor lung function received nebulized treatment with drugs such as budesonide and underwent respiratory function exercises to optimize their respiratory function. Preoperative anesthesia consultation followed the American Society of Anesthesiologists (ASA) physical status classification for patient assessment, ensuring their suitability for general anesthesia. The skin in the surgical area was prepared and cleaned one day before the surgery, with hair removal performed in an area of at least 15 cm around the incision, and patients were instructed to refrain from eating and drinking as per standard protocol.

### 2.4. Surgical procedures

#### 2.4.1. Laminotomy and decompression surgery

**Figure 1.**
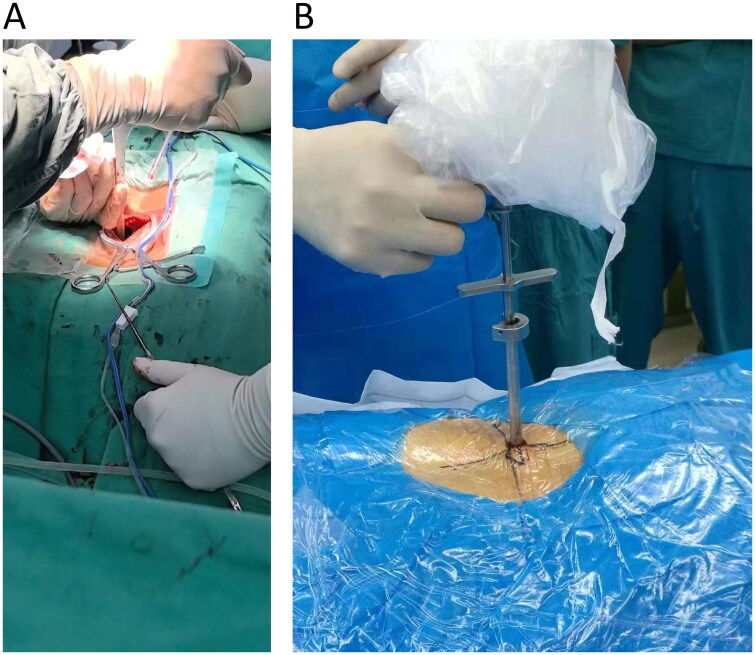
Intraoperative photos of decompression by laminotomy surgery (A) and percutaneous tansforaminal endoscopic surgery (B).

Following successful general anesthesia induction, patients were placed in a prone position, and standard disinfection procedures were performed. Using fluoroscopy to identify the responsible intervertebral space, a 15 cm midline incision was made, and the skin, subcutaneous fat, and lumbodorsal fascia were sequentially dissected. The sacrospinal muscle was detached to expose the lamina gap. Partial laminectomy and hypertrophied ligamentum flavum resection were performed, along with the removal of hypertrophic and internally fused articular processes to decompress the dura mater and nerve roots (Figure 1A). In cases of significant intervertebral disc protrusion, the protruded and degenerated nucleus pulposus tissue was excised. The extent and degree of decompression were determined intraoperatively based on the surgeon’s experience.

#### 2.4.2. Percutaneous transforaminal endoscopic spinal canal decompression surgery

The patient is placed in a prone position, and the C-arm X-ray machine is used for target intervertebral space localization. Routine disinfection and draping are performed, and 0.1% lidocaine local anesthesia is administered. A puncture needle is inserted approximately 10-14 cm lateral to the midline of the symptomatic side of the target spinous process under fluoroscopic monitoring at a 10° angle to the horizontal plane. The needle is advanced through the safe zone of the intervertebral foramen (Kambin’s triangle) into the spinal canal. Soft tissue is gradually dilated using 1-4 grade expansion tubes, and a ring saw is used to grind the ventral side of the lower vertebral body articular process. If necessary, further grinding may be required on the lower part of the articular process and the upper margin and inner edge of the lower vertebral arch to enlarge the intervertebral foramen and nerve root canal. A working channel is created, and an optical source and lens are connected, with the screen image adjusted. The lens is directly inserted into the spinal canal for radiofrequency hemostasis, excision of proliferative yellow ligament tissue around the nerve root, exploration of the protruding intervertebral disc, and under microscope guidance, resection of the protruding intervertebral disc tissue and partial proliferative ligament tissue to relieve nerve root compression. Clear pulsation of the dura mater and nerve roots is observed after confirmation of no ­compression, followed by irrigation of the incision, radiofrequency ablation decompression, and annulus fibrosus shaping (Figure 1B). The working channel is removed, the incision is sutured, dressings are applied, and the procedure is concluded.

### 2.5. Observation indicators

#### 2.5.1. Pain assessment

The preoperative and postoperative pain levels of the patients were assessed using the Visual Analog Scale (VAS) for pain scores, ranging from 0 to 10 points, where a higher score indicates more severe pain.

#### 2.5.2. Lumbar spine function

The Oswestry Disability Index (ODI) questionnaire consists of 10 questions, covering aspects such as pain intensity, self-care, lifting, walking, sitting, standing, sleep disturbance, sex life, social life, and traveling. Each question has 6 options, with a maximum score of 5 for each question. Choosing the first option scores 0, and the last option scores 5. If all 10 questions are answered, the scoring method is: actual score/50 (maximum possible score) x 100%. If one question is left unanswered, the scoring method is: actual score/45 (maximum possible score) x 100%. A higher score indicates more severe functional impairment.

The Japanese Orthopaedic Association Back Pain Evaluation Questionnaire (JOA) encompasses subjective symptoms, activities of daily living, clinical signs, and bladder function, with scores of 9, 14, 6, and −6 to 0, respectively, and a maximum score of 29. A higher score implies a better functional status.

### 2.6. Data collection

Collect demographic data, preoperative clinical characteristics, surgical specifics, postoperative complications, wound healing status, pain intensity measurements (utilizing the Visual Analog Scale), and assess lumbar function using standardized scoring systems such as the Japanese Orthopaedic Association (JOA) and Oswestry Disability Index (ODI). The wound healing condition is judged by the doctor, and there are no uncomfortable symptoms such as redness, swelling, exudation, and pain at the wound, which indicates that the tissue on the surface and under the wound is basically healed.

### 2.7. Statistical analysis

The data were analyzed using SPSS 25.0 statistical software (SPSS Inc., Chicago, IL,USA). For categorical data, [n(%)] was used for representation. The chi-square test was applied with the basic formula when the sample size was ≥40 and the theoretical frequency T was ≥5, with the test statistic represented by χ2. When the sample size was ≥40 but the theoretical frequency 1≤*T* < 5, the chi-square test was adjusted using the correction formula. For normally distributed continuous data, the format (X ± s) was employed. Non-normally distributed data was analyzed using Wilcoxon rank-sum test. *p* < 0.05 were considered as statistical significance.

## 3. Results

### 3.1. General information

Based on the results of our study comparing the influence of decompression by laminotomy and percutaneous transforaminal endoscopic surgery on postoperative wound healing, pain intensity, and lumbar function in elderly patients with lumbar spinal stenosis, there were no significant differences between the laminotomy group (*n* = 65) and the percutaneous transforaminal endoscopic group (*n* = 69) with respect to age, gender distribution, body mass index (BMI), smoking status, alcohol consumption, diabetes prevalence, hypertension prevalence, hyperlipidemia prevalence, history of lumbar injury, physical labor intensity, and the course of the disease (*p* > 0.05). Our study found no significant differences between the two groups in terms of the distribution of different conditions, including pure lumbar spinal stenosis (21.54% vs. 21.74%), lumbar disc herniation (49.23% vs. 50.72%), degenerative spondylolisthesis (26.15% vs. 23.19%), and degenerative scoliosis (3.08% vs. 4.35%). Similarly, there were no significant differences in the distribution of ASA Classification grades between the two groups, with Grade I (12.31% vs. 14.49%), Grade II (76.92% vs. 71.01%), and Grade III (10.77% vs. 14.49%). There were no significant differences observed in the distribution of stenosis at L2-3 (4.62% vs. 7.25%), L3-4 (23.08% vs. 20.29%), and L4-5 (44.62% vs. 47.83%) levels. However, at the L5-S1 level, there was a trend towards a higher incidence of stenosis in the percutaneous transforaminal endoscopic group compared to the laminotomy group (27.69% vs. 24.64%), although this did not reach statistical significance (*t* = 0.042, *p* = 0.837). The comparison of surgical parameters between the laminotomy group and the percutaneous transforaminal endoscopic group revealed significant differences in surgical time and intraoperative blood loss. Specifically, the percutaneous transforaminal endoscopic surgery demonstrated significantly reduced surgical time compared to laminotomy (70.78 ± 6.80 min vs. 128.97 ± 4.70 min, *t* = 4485, *p* < 0.001), along with substantially decreased intraoperative blood loss (94.22 ± 7.69 mL vs. 327.68 ± 6.44 mL, *t* = 190.871, *p* < 0.001). However, no significant difference was found in the length of hospital stay between the two groups (14.26 ± 5.45 days vs. 13.49 ± 2.49 days, *t* = 1.060, *p* = 0.292). The study demonstrated comparable baseline demographic and clinical characteristics between the two groups, which strengthens the validity of the subsequent comparisons for the primary outcome measures (Table 1).

**Table 1.** General Information and demographic characteristics of patients.

Parameters	Decompression by Laminotomy group (*n* = 65)	Percutaneous Tansforaminal Endoscopic group (*n* = 69)	t/χ^2^	P
Age (years)	69.77 ± 5.74	69.96 ± 4.62	0.207	0.836
Gender			0.298	0.585
Male	28 (43.08%)	34 (49.28%)		
Female	37 (56.92%)	35 (50.72%)		
BMI			0.030	0.863
<24	17 (26.15%)	20 (28.99%)		
>24	48 (73.85%)	49 (71.01%)		
Smoking	19 (29.23%)	19 (27.54%)	0.001	0.979
Drinking	20 (30.77%)	23 (33.33%)	0.018	0.894
Diabetes	15 (23.08%)	13 (18.84%)	0.152	0.696
Hypertension	16 (24.62%)	14 (20.29%)	0.154	0.694
Hyperlipidemia	11 (16.92%)	10 (14.49%)	0.022	0.882
History of Lumbar Injury	6 (9.23%)	8 (11.59%)	0.027	0.869
Physical Labor Intensity			0.933	0.334
Light to Moderate	43 (66.15%)	39 (56.52%)		
Severe	22 (33.85%)	30 (43.48%)		
Course of Disease			5.357	0.021*
<1 year	44 (67.69%)	32 (46.38%)		
≥1 year	21 (32.31%)	37 (53.62%)		
Condition			0.280	0.964
Pure Lumbar Spinal Stenosis	14 (21.54%)	15 (21.74%)		
Lumbar Disc Herniation	32 (49.23%)	35 (50.72%)		
Degenerative Spondylolisthesis	17 (26.15%)	16 (23.19%)		
Degenerative Scoliosis	2 (3.08%)	3 (4.35%)		
ASA Classification			0.643	0.725
Grade I	8 (12.31%)	10 (14.49%)		
Grade II	50 (76.92%)	49 (71.01%)		
Grade III	7 (10.77%)	10 (14.49%)		
Lumbar Spinal Stenosis Levels				
L2-3	3 (4.62%)	5 (7.25%)	0.077	0.781
L3-4	15 (23.08%)	14 (20.29%)	0.033	0.856
L4-5	29 (44.62%)	33 (47.83%)	0.040	0.842
L5-S1	18 (27.69%)	17 (24.64%)	0.042	0.837
Surgical Time (min)	128.97 ± 4.70	70.78 ± 6.80	4485.000	p < 0.001
Intraoperative Blood Loss (mL)	327.68 ± 6.44	94.22 ± 7.69	190.871	p < 0.001
Length of Hospital Stay (days)	14.26 ± 5.45	13.49 ± 2.49	1.060	0.292

Note: *, p less than 0.05 indicated that there was a significant difference between the two groups, and the difference was statistically significant.

### 3.2. Postoperative complications and evaluation scores

The comparison of complications between the laminotomy group and the percutaneous transforaminal endoscopic group did not reveal any statistically significant differences in the incidence of dural tear (1.54% vs. 1.45%), urinary tract infection (6.15% vs. 0%), urinary retention (6.15% vs. 0%), pneumonia (3.08% vs. 2.90%), and postoperative anemia (1.54% vs. 1.45%). The comparison of postoperative wound healing between the two group revealed significant differences in ambulation time and wound healing time. The percutaneous transforaminal endoscopic surgery demonstrated a markedly shorter ambulation time compared to laminotomy (3.00 ± 0.00 days vs. 5.06 ± 0.30 days, χ2 = 134, *p* < 0.001). Similarly, the wound healing time was significantly shorter in the percutaneous transforaminal endoscopic group compared to the laminotomy group (9.93 ± 1.19 days vs. 12.23 ± 1.74 days, *W* = 3873, *p* < 0.001). In the comparison of pain intensity before and after surgery between thetwo groups, no statistically significant differences were observed in preoperative pain levels (VAS) (7.29 ± 1.17 vs. 7.46 ± 1.12, *p* = 0.758). However, postoperative pain levels (VAS) were found to be significantly lower in the percutaneous transforaminal endoscopic surgery group compared to the laminotomy group (3.48 ± 1.11 vs. 2.80 ± 1.05, *p* = 0.007). In comparing the JOA scores between the laminotomy group and the percutaneous transforaminal endoscopic surgery group, no statistically significant differences were found in the pre-treatment scores for lower back pain, leg pain, walking ability, and total JOA scores. However, post-treatment JOA scores showed significantly greater improvement in the percutaneous transforaminal endoscopic surgery group compared to the laminotomy group for lower back pain (2.09 ± 0.28 vs. 1.92 ± 0.48, **χ2 **=** **11.477, *p* = 0.003), leg pain (2.00 ± 0.00 vs. 1.86 ± 0.66, **χ2 **=** **34.244, *p* < 0.001), walking ability (2.33 ± 0.47 vs. 1.88 ± 0.74, χ2 = 25.194, *p* < 0.001), and total JOA (22.87 ± 2.43 vs. 21.82 ± 3.13 *W* = 2.170, *p* = 0.032). In comparing the ODI scores between the two groups, no statistically significant differences were found in the pre-treatment scores (70.85 ± 0.48vs. 70.70 ± 0.52, *t* = 2558.5, *p* = 0.081). However, post-treatment ODI scores demonstrated a significant improvement in the percutaneous transforaminal endoscopic surgery group compared to the laminotomy group (37.94 ± 0.50 vs. 35.06 ± 0.29, *t* = 4485, *p* < 0.001) (Table 2).

**Table 2.** Postoperative complications and evaluation scores in the two groups.

Parameters	Decompression by Laminotomy group (*n* = 65)	Percutaneous Tansforaminal Endoscopic group (*n* = 69)	t/χ2/W	P
Complications				
Dural tear	1 (1.54%)	1 (1.45%)	0	1
Urinary tract infection	4 (6.15%)	0 (0.00%)	2.510	0.113
Urinary retention	4 (6.15%)	0 (0.00%)	2.510	0.113
Pneumonia	2 (3.08%)	2 (2.90%)	0	1
Postoperative anemia	1 (1.54%)	1 (1.45%)	0	1
Wound Healing				
Ambulation Time (d)	5.06 ± 0.30	3.00 ± 0.00	134.000	p < 0.001*
Wound Healing Time(d)	12.23 ± 1.74	9.93 ± 1.19	3873.000	p < 0.001*
Pain Intensity				
VAS				
Before Treatment	7.29 ± 1.17	7.46 ± 1.12	2.620	0.758
After Treatment	3.48 ± 1.11	2.80 ± 1.05	17.614	0.007*
JOA Scores				
Lower Back Pain				
Before Treatment	0.82 ± 0.50	0.68 ± 0.56	2.792	0.248
After Treatment	1.92 ± 0.48	2.09 ± 0.28	11.477	0.003*
Leg Pain				
Before Treatment	0.71 ± 0.46	0.58 ± 0.50	1.860	0.173
After Treatment	1.86 ± 0.66	2.00 ± 0.00	34.244	p < 0.001
Walking Ability				
Before Treatment	1.11 ± 0.50	1.12 ± 0.47	1.349	0.718
After Treatment	1.88 ± 0.74	2.33 ± 0.47	25.194	p < 0.001
Total				
Before Treatment	15.55 ± 3.15	15.91 ± 2.88	0.688	0.493
After Treatment	21.82 ± 3.13	22.87 ± 2.43	2.170	0.032*
ODI Scores				
Before Treatment	70.85 ± 0.48	70.70 ± 0.52	2558.500	0.081
After Treatment	37.94 ± 0.50	35.06 ± 0.29	4485.000	p < 0.001

Note: *, p less than 0.05 indicated that there was a significant difference between the two groups, and the difference was statistically significant.

### 3.3. Correlation analysis

Based on the correlation analysis, significant negative correlations were observed between surgical time, intraoperative blood loss, and ambulation time with postoperative wound healing, pain intensity, and lumbar spine function in elderly patients with lumbar spinal stenosis (r = −0.981, r2 = 0.962, *p* < 0.001; r = −0.998, r2 = 0.996, *p* < 0.001; r = −0.979, r2 = 0.959, *p* < 0.001, respectively). However, the correlation between wound healing time and postoperative pain, lower back pain, leg pain, walking ability, total Japanese Orthopaedic Association (JOA) score, and Oswestry Disability Index (ODI) scores after treatment did not reach statistical significance (*p* > 0.05). Notably, the correlation coefficients for postoperative pain, lower back pain, leg pain, walking ability, total JOA score, and ODI scores after treatment were 0.251, 0.197, 0.297, 0.304, 0.186, and −0.97, respectively, with corresponding r2 values of 0.063, 0.039, 0.088, 0.092, 0.035, and 0.941. Findings suggest important associations between surgical variables and postoperative outcomes in elderly patients with lumbar spinal stenosis (Table 3).

**Table 3.** Correlation analysis of surgical approach with postoperative wound healing, pain intensity, and lumbar spine function in elderly patients with lumbar spinal stenosis.

Parameters	r	R^2^	P
Surgical Time	−0.981	0.962	p < 0.001*
Intraoperative Blood Loss	−0.998	0.996	p < 0.001*
Ambulation Time	−0.979	0.959	p < 0.001*
Wound Healing Time	−0.632	0.4	p < 0.001*
Postoperative Pain	−0.251	0.063	0.003*
Lower Back Pain (After Treatment)	0.197	0.039	0.023*
Leg Pain (After Treatment)	0.297	0.088	p < 0.001*
Walking Ability (After Treatment)	0.304	0.092	p < 0.001*
Total JOA (After Treatment)	0.186	0.035	0.031*
ODI Scores (After Treatment)	−0.97	0.941	p < 0.001*

Note: *, p less than 0.05 indicated that there was a significant difference between the two groups, and the difference was statistically significant.

## 4. Discussion

Lumbar spinal stenosis was a prevalent condition in the elderly population, often leading to significant impairment in physical function and quality of life [16]. Surgical intervention becomes necessary when conservative treatments fail to alleviate symptoms [17,18]. This study aimed to investigate the impact of laminotomy decompression and percutaneous transforaminal endoscopic treatment on postoperative wound healing, pain intensity, and lumbar function in elderly patients with lumbar spinal stenosis.

Surgical parameters played a significant role in differentiating the two surgical approaches [12]. Percutaneous transforaminal endoscopic surgery demonstrated significantly shorter surgical time and reduced intraoperative blood loss compared to laminotomy. These findings align with the advantages typically associated with minimally invasive surgical techniques, suggesting that the minimally invasive nature of percutaneous transforaminal endoscopic surgery allows for smaller incisions and reduced tissue disruption, leading to shorter surgical time and reduced intraoperative blood loss [19,20]. This approach relies on advanced endoscopic techniques and specialized instruments to access the spinal canal and perform decompression with minimal disruption to surrounding tissues [21,22].

There were no significant differences in the incidence of dural tear, pneumonia, and postoperative anemia between the two surgical methods. However, the Laminotomy group showed a significantly higher incidence of urinary tract infection and urinary retention compared to the Decompression by Percutaneous Transforaminal Endoscopic group. However, the difference was not statistically significant. These findings suggest that percutaneous endoscopic lumbar surgery is relatively safe, highlighting its feasibility in treating elderly patients with lumbar spinal stenosis. Because the doctors and nurses were very experienced in Decompression by Laminotomy Surgeryand Percutaneous Tansforaminal Endoscopic Surgery, and the postoperative care was very good, this made the incidence of postoperative complications very low, and there was a certain difference in the number of cases between the two groups, but the difference was not statistically significant. In recent years, there have been many studies on the treatment of lumbar spinal stenosis in the elderly, and the results of these studies were consistent with ours. In this study, the postoperative complications of patients with lumbar spine surgery showed that Urinary tract infection and Urinary retention occurred in 4 cases (6.15%) and 4 cases (6.15%) respectively in Decompression by Laminotomy group (*n* = 65). However, Urinary tract infection and Urinary retention were not found in Percutaneous Tansforaminal Endoscopic group (*n* = 69). Obviously Percutaneous cutaneous Tansforaminal Endoscopic surgery has advantages, but our statistical results showed that the p of both comparisons was 0.113, which may indicate that there is an error in our study. Although there are some errors in our study, the results of our study are similar to those of other recent studies, many of which have shown that patients with Urinary tract infection or Urinary retention after lumbar surgery are generally poor in health or have had multiple lumbar surgeries. Patil A et al. [23] performed laminectomy in patients with achondroplasia and hypoplasia and followed up 90 days after surgery to compare the different types of adverse events. Patients with achondroplasia had significantly higher rates of blood transfusion (OR = 6.40, *p* < 0.001), urinary tract infection (OR = 3.79, *p* < 0.001), wound rupture (OR = 3.71, *p* < 0.001), and hematoma (OR = 2.94, *p* = 0.032). Mormol JD et al. [24] studied the risk factors of urinary retention after posterior lumbar fusion. A retrospective cohort study was conducted on the characteristics of 814 patients who underwent lumbar laminectomy and fusion before, during and after surgery, and the results showed that postoperative urinary tract infection (OR = 5.60, *p* = 0.005) was associated with postoperative urinary retention. History of previous lumbar surgery (OR = 0.55; *p* = 0.019) was associated with decreased urinary retention after surgery.

Postoperative wound healing and pain intensity were notable areas of divergence between the two surgical approaches. Percutaneous transforaminal endoscopic surgery demonstrated significantly shorter ambulation time and wound healing time compared to laminotomy. Additionally, postoperative pain levels were significantly lower in the percutaneous transforaminal endoscopic group, highlighting the benefits of this minimally invasive approach in promoting faster recovery and improved pain control in elderly patients with lumbar spinal stenosis. The use of percutaneous transforaminal endoscopic surgery involves targeted visualization and precise manipulation of anatomical structures, allowing for more focused and efficient decompression of neural elements [25,26]. By specifically targeting the affected area through a transforaminal approach, the procedure may minimize the need for extensive tissue retraction and bony removal, contributing to faster wound healing and reduced postoperative pain [27,28].

The functional outcomes, as assessed by JOA and ODI scores, revealed interesting trends favoring percutaneous transforaminal endoscopic surgery. Post-treatment JOA scores showed significantly greater improvement in the percutaneous transforaminal endoscopic group for lower back pain, leg pain, and walking ability. Similarly, post-treatment ODI scores demonstrated significant improvement in the percutaneous transforaminal endoscopic group compared to the laminotomy group. These findings suggest the potential effectiveness of percutaneous transforaminal endoscopic surgery in achieving improved postoperative pain intensity and lumbar function in elderly patients with lumbar spinal stenosis, supporting its consideration as a favorable treatment modality in this patient population. The reduced tissue trauma associated with percutaneous transforaminal endoscopic surgery may lead to less postoperative inflammation and scarring, which were crucial factors in promoting faster wound healing and overall recovery [29,30]. Furthermore, the minimally invasive approach may result in better preservation of surrounding musculature, potentially leading to enhanced postoperative lumbar function and reduced postoperative pain [31,32].

The correlation analysis in our study revealed important associations between surgical variables and postoperative outcomes in elderly patients with lumbar spinal stenosis. Specifically, surgical time, intraoperative blood loss, and ambulation time showed significant negative correlations with postoperative wound healing, pain intensity, and lumbar spine function. These findings emphasize the importance of surgical variables in influencing postoperative outcomes and highlight the potential benefits of minimizing surgical time and intraoperative blood loss in achieving favorable postoperative results in this patient population.

The results of this study contribute valuable data to the existing literature by providing comparative insights into the outcomes of laminotomy decompression and percutaneous transforaminal endoscopic treatment in elderly patients with lumbar spinal stenosis. These findings support the consideration of percutaneous transforaminal endoscopic surgery as a promising alternative to traditional laminotomy decompression in this patient population.

Nevertheless, it was important to acknowledge certain limitations of this study. Firstly, the retrospective nature of the study may have introduced inherent biases and limitations in data collection. At the same time, the grouping process is not random, but made according to the doctor’s advice and considering the patient’s economic situation, which is prone to selectivity bias. Additionally, the relatively limited sample size may impact the generalizability of the findings. Future prospective studies with larger sample sizes and longer follow-up periods were warranted to further validate these results and provide more robust evidence regarding the comparative effectiveness of the two surgical approaches in elderly patients with lumbar spinal stenosis.

## 5. Conclusion

In conclusion, this study provides valuable insights into the influence of laminotomy decompression and percutaneous transforaminal endoscopic treatment on postoperative outcomes in elderly patients with lumbar spinal stenosis. The findings suggest that percutaneous transforaminal endoscopic surgery may offer advantages in terms of shorter surgical time, reduced intraoperative blood loss, faster wound healing, improved pain control, and enhanced lumbar function compared to traditional laminotomy decompression. These results warrant further consideration and prospective investigation to guide clinical decision-making and improve outcomes for elderly patients with lumbar spinal stenosis.

## Data Availability

The datasets used and/or analyzed during the current study are available from the corresponding author on reasonable request.
